# Maternal and perinatal outcomes of pregnancy associated with COVID-19: Systematic review and meta-analysis

**DOI:** 10.18332/ejm/149485

**Published:** 2022-07-06

**Authors:** Zekiye Karaçam, Damla Kizilca-Çakaloz, Gizem Güneş-Öztürk, Ayden Çoban

**Affiliations:** 1 Department of Midwifery, Faculty of Health Sciences, Aydın Adnan Menderes University, Aydın, Turkey

**Keywords:** health care, pregnancy outcome, maternal mortality, COVID-19, baby health

## Abstract

**INTRODUCTION:**

This study explored maternal and infant outcomes in the periods of pregnancy, birth and the postpartum, in women with COVID-19.

**METHODS:**

After PROSPERO registration (CRD42020191106), scanning for the studies was carried out over the period 5–15 May 2020 in the PubMed, Science Direct, EBSCO and Web of Science databases with the search string: [‘COVID-19’ AND (‘pregnancy’ OR ‘pregnant’ OR ‘maternal outcomes’ OR ‘infant outcomes’ OR ‘fetal outcomes’ OR ‘birth’)]. Studies reporting maternal and perinatal outcomes of pregnant women with COVID-19 were included. Data were extracted independently by two researchers and combined with meta-analysis and pooled analysis.

**RESULTS:**

The 54 studies included in this analysis contained data on 517 pregnant women diagnosed with COVID-19 and 385 infants. Of the pregnant women, 18% had gone into preterm labor and 77% had given birth by caesarean. Of the newborns, 19% had low birth weight, 14% had fetal distress, and 24% were admitted into the neonatal intensive care unit. Nine maternal and eight baby mortalities were reported in the studies.

**CONCLUSIONS:**

The study revealed that COVID-19 in pregnant women appeared to be negative maternal and infant outcomes, with mortalities as well.

## INTRODUCTION

In February 2020, the World Health Organization (WHO) reported the rapidly spreading global outbreak of a new (novel) coronavirus disease, COVID-19, that had appeared in the last part of 2019 in the city of Wuhan, China. WHO announced that COVID-19 was a pandemic that was an international threat and a global public health emergency^1^. Over the last two years, this pandemic has caused significant changes in the daily life of people, especially in education and working conditions, in addition to mortality and morbidity rates. During this period, significant progress has been made in the fight against the pandemic with effective health policies, lifestyle changes and widespread vaccination programs. However, especially due to the emergence of new variants, COVID-19 has still not been fully controlled. Efforts to control the pandemic have caused unprecedented economic and social disruption all over the world^2,3^.

The immune system of the pregnant woman, the changes in her cardiopulmonary, respiratory and physiological systems put her at high risk in terms of becoming infected with respiratory viruses and developing more severe disease. Studies conducted so far have not provided any evidence that pregnant women are any more susceptible to COVID-19 than others^4–6^. However, the course of disease seems to be worse in pregnant women compared to non-pregnant women of the same age. Although more than 90% of pregnant women with COVID-19 recover without serious morbidity, rapid deterioration of disease may be observed; especially symptomatic pregnant women have a higher risk for severe disease compared to the symptomatic non-pregnant women with COVID-19^2,7,8^.

Current studies to date have shown that pregnant women with COVID-19 are at higher risk for serious illness, need for mechanical ventilation, admission to the intensive care unit, and maternal death. In addition, obstetric complications such as preeclampsia, preterm birth, and premature rupture of membranes, fetal distress, and stillbirth are more common in pregnancies complicated by COVID-19^9,10^. Moreover, some recent publications show that pregnant women infected with new SARS-CoV-2 variants have a worse prognosis^11,12^. In a study comparing pre-variant and post-variant pregnant groups in Turkey, it was reported that there was a significant increase in the rates of serious and critical cases and in the rates of pregnancy complications, preterm delivery, respiratory support, intensive care unit admission and maternal death, and admission to NICU in the post-variant group^3^. Although there is evidence of intrauterine transmission of SARS-CoV-2, it is reported to occur rarely^2^.

Health professionals have an important responsibility in the periods of pregnancy, birth and the postpartum, not only to provide follow-up and care for both mother and child but also to offer preventive, early diagnosis, treatment and individualized care services^13-15^. Vaccination appears to be one of the most effective means of protection in the COVID-19 pandemic. COVID-19 vaccines approved by the U.S. Food and Drug Administration can be administered to pregnant or lactating women^2^. In the management process of the COVID-19 pandemic, health professionals need to manage the perinatal period more effectively, using a range of comprehensive knowledge to protect and improve maternal and infant health. They can carry out their follow-up and care services in the perinatal process in accordance with the guidelines published by both national and international sources^13-15^, thus contributing to the production of more comprehensive scientific knowledge.

During the planning and execution of this study, a look into the literature brings forth systematic reviews, case reports, case series and retrospective cross-sectional international studies conducted with small samples that report perinatal outcomes for pregnant women with COVID-19. However, two systematic review studies have recently been published covering approximately the same time as our study. In these systematic reviews, important information about the effects of COVID-19 infection on mother–infant health has been revealed^9,10^. On the other hand, there is still an urgent need for high quality evidence-backed scientific studies that will disclose broader sets of data on the subject. It was for this reason that this systematic review and meta-analysis was conducted to examine the maternal and infant health outcomes of pregnant women with COVID-19 during the pregnancy, birth and postpartum periods.

## METHODS

### Protocol and registration

The protocol of this systematic review and meta-analysis was registered on the PROSPERO database (CRD42020191106; registered 11 June 2020). The review and its reporting were followed up on PRISMA (Preferred Reporting items for Systematic Review and Meta-Analysis)^16^. To keep the risk of bias under control during the study, the scanning, selection of articles, data extraction as well as the quality assessment of the articles included were handled independently by two researchers. Whenever a difference of opinion came up with regard to any aspect of the study, all of the researchers held a discussion session together and arrived at an agreement. At the same time, prior to the start of the study, a pilot study that included all the stages of the research was conducted with the participation of all of the authors, who concurred on a common road map.

### Eligibility criteria

The research included those studies published in English between 1 December 2019 and 15 May 2020 and whose full texts could be accessed. The PICOS criteria were considered in the selection of the studies that would be suitable for this systematic review and meta-analysis (Table 1). The exclusion criteria encompassed traditional and systematic reviews and letters to the editor that did not refer to specific cases.

**Table 1:** PICOS criteria for inclusion of studies and data extraction

*Parameters (PICOS)*	*Inclusion criteria*	*Extraction criteria*
**Population**	Pregnant women with COVID-19	Country, age
**Exposure**	COVID-19	COVID-19 test outcomes
**Comparators**	None	-
**Outcomes**		
Data on COVID-19	Presence of data on pregnant women	Symptoms of COVID-19, comorbidities, methods of diagnosis and treatment
Data on pregnancy	Presence of data on pregnancy	Gestation period (weeks), preterm labor, delivery mode, caesarean indications, pregnancy-related disorders, admission to the intensive care unit, mechanical ventilation, postpartum health status, explanation of the care process, maternal death
Data on baby’s health	Presence of data on newborns	Birth weight, premature, low birth weight, APGAR score 1 and 5 min, fetal distress, asphyxia, NICU acceptance, feeding, fetal anomaly, intrauterine and neonatal death, IgG, IgM, RT-PCR test and chest radiograph outcomes, follow-up and the methods used in its maintenance
**Study design**	Case reports, case series, case-control studies, retrospective and prospective observational studies	Design of studies

### Searching strategy

The scanning for this systematic review and meta-analysis was carried out over the period 5–15 May 2020 independently by two of the researchers. Scanning for the international studies was carried out in the PubMed, Ovid, Science Direct, EBSCO and Web of Science, Google Scholar electronic databases with the search string: [‘COVID-19’ AND (‘pregnancy’ OR ‘pregnant’ OR ‘maternal outcomes’ OR ‘infant outcomes’ OR ‘fetal outcomes’ OR ‘birth’)]. At the same time, additional studies were independently checked by the other two authors against the included articles and the reference lists for the review studies.

### Selection of studies

**Figure 1:**
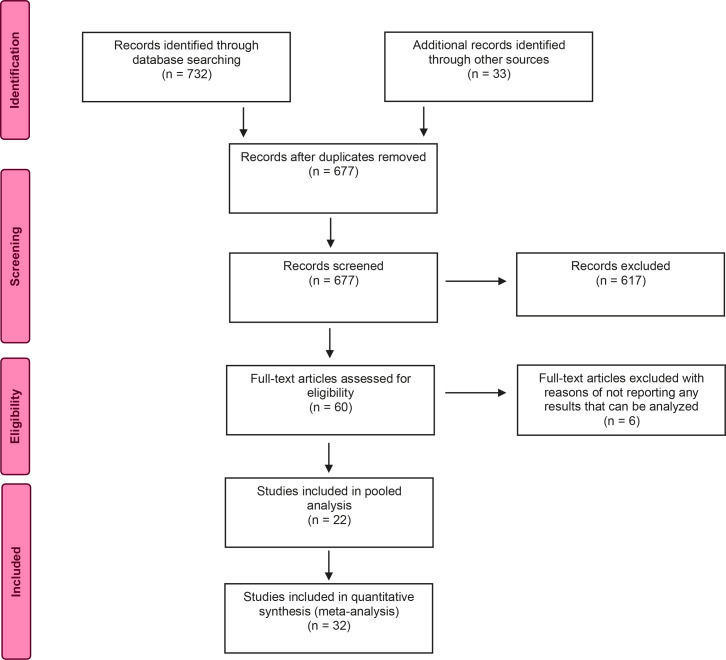
PRISMA flow diagram of the search process

Through the processes of scanning, selection according to heading and/or abstract, and the elimination of repetitious articles. The four authors then met together to decide upon the studies that could be taken into the analysis on the basis of full texts. Later, some studies were eliminated from the scope of the analysis during the extraction or analysis processes because they did not contain data appropriate for the analysis. The selection process for the articles can be seen in Figure 1 with a PRISMA flow diagram.

### Data extraction

The researchers devised a data extraction instrument to be used in obtaining study data (Table 2). This tool made it possible to collect information on the studies included in the systematic review and meta-analysis in terms of author information, the country and year of publication, data collection dates, design, sample size, maternal age and other main findings reported in the studies (Table 1). In four studies of case-control design, only the data pertaining to the cases (pregnant women diagnosed with COVID-19) were extracted.

**Table 2:** Characteristics and main findings of the studies included in the systematic review and meta-analyses

*Study and Country*	*Date of data collection*	*Study design*	*Sample size Age (years)*	*Data on COVID-19*	*Data on pregnancy, birth and maternal health*	*Data on baby’s health*	*Quality score*
Alzamora et al.^58^ Peru	29 March 2020	Case report	n=1 44	Symptoms, comorbidities, diagnostic test, treatment protocol	Gestation period (weeks), mode of delivery, preterm delivery	Birth weight, 1 and 5 min APGAR score, intubation NICU admission, IgG, IgM and RT-PCR test outcomes, breastfeeding	Yes: 7/8
Breslin et al.^29^ USA	13–27 March 2020	Retrospective	n=43 Mean: 29.7±6.0	Symptoms, comorbidities, diagnostic test, treatment protocol	Gestational period (weeks), preterm delivery, pregnancy-related disorders, mode of delivery, caesarean indications, ICU admission, antepartum and postpartum hospitalization and treatment protocol, explanation of the care process	1 and 5 minutes APGAR score, NICU admission, prematurity, additional diagnoses, RT-PCR test outcomes, breastfeeding	Yes: 6/8
Browne et al.^18^ Colombia	-	Case report	n=1 33	Symptoms, comorbidities, diagnostic test, treatment protocol	Gestational period (weeks), preterm delivery, pregnancy-related disorders, explanation of the care process	-	Yes: 7/8
Buonsenso et al.^19^ Italy	-	Case series	n=2 38 and 42	Symptoms, diagnostic test, treatment protocol	Gestation period (weeks), mode of delivery, explanation of the care process	Birth weight, 1 and 5 min APGAR score, fetal distress, NICU admission, IgG, IgM and RT-PCR test outcomes, breastfeeding	Yes: 9/10
Cao et al.^37^ China	23 January – 23 February 2020	Retrospective	n=10 (Twins: 1) 29 (n=4), 30 (n=3), 31 (n=2), 35 (n=1)	Symptoms, diagnostic test	Gestational period (weeks), preterm delivery, pregnancy-related disorders, mode of delivery, caesarean indications, explanation of the care process	Birth weight, prematurity, 1 and 5 min APGAR score, fetal distress, neonatal asphyxia, neonatal death, RT-PCR test outcomes	Yes: 6/8
Chen et al.^38^ China	20–31 January 2020	Retrospective	n=9 26 (n=3), 27, 28, 29, 33, 34, 40	Symptoms, comorbidities, diagnostic test	Gestational period (weeks), preterm delivery, pregnancy-related disorders, mode of delivery	Birth weight, low birth weight, prematurity, 1 and 5 min APGAR score, fetal distress, neonatal asphyxia, intrauterine and neonatal death, RT-PCR test outcomes	Yes: 6/8
Chen et al.^39^ China	8 December 2019 – 20 March 2020	Retrospective	n=118 Median 31 (range: 28-34) (Twins: 2)	Symptoms, diagnostic test, treatment protocol	Preterm delivery, maternal death, mode of delivery, caesarean indication, abortion	1 min APGAR score, neonatal death, neonatal asphyxia; RT-PCR test outcomes	Yes: 6/8
Chen et al.^56^ China	20 January – 10 February 2020	Case series	n=5 25, 29 (n=2), 30, 31	Symptoms, diagnostic tests	Gestational period (weeks), pregnancy-related disorders, mode of delivery, postpartum health status	Birth weight, 5 min APGAR score, fetal tachycardia, RT-PCR test outcomes, feeding	Yes: 10/10
Chen et al.^40^ China	22–28 February 2020	Case series	n=4 23, 28, 31, 34	Symptoms, comorbidities, diagnostic test	Gestational period (weeks), pregnancy-related disorders, mode of delivery, explanation of the care process, postpartum health status	Birth weight, 1 and 5 min APGAR score, fetal distress, NICU admission, RT-PCR test outcomes, feeding	Yes: 9/10
Dong et al.^59^ China	28 January –28 February 2020	Case report	n=1 29	Symptoms, diagnostic test, treatment protocol	Gestational period (weeks), mode of delivery, explanation of the care process	Birth weight, 1 and 5 min APGAR score, NICU admission, IgG, IgM and RT-PCR test outcomes	Yes: 7/8
Fan et al.^57^ China	10 January –27 February 2020	Case series	n=2 29, 34	Symptoms, diagnostic test, treatment protocol	Gestational period (weeks), mode of delivery, explanation of the care process	Birth weight, 1 and 5 min APGAR score, RT-PCR and Chest CT test outcomes, feeding	Yes: 9/10
Ferrazzia et al.^41^ Italy	1–20 March 2020	Retrospective	n=42 (21–44)	Symptoms, diagnostic test, treatment protocol	Gestational period (weeks), pregnancy-related disorders, ICU admission, mode of delivery, caesarean indications, postpartum health status, explanation of the care process	Birth weight, 5 min APGAR score, NICU admission, RT-PCR test outcomes, feeding	Yes: 6/8
Gidlof et al.^20^ Sweden	-	Case report	n=1 (Twins: 1) 34	Symptoms, comorbidities, diagnostic test, treatment protocol	Gestational period (weeks), pregnancy-related disorders, mode of delivery, explanations of the care process	Birth weight, low birth weight, 1 and 5 min APGAR score, NICU admission, RT-PCR test outcomes, feeding	Yes: 7/8
Govind et al.^42^ United Kingdom	7 March – 22 April 2020	Case series	n=9 median: 31 (range: 18–39)	Symptoms, diagnostic test, comorbidities	Gestational period (weeks), preterm delivery, pregnancy-related disorders, mode of delivery, caesarean indications, explanations of the care process	Birth weight, low birth weight, 1 and 5 min APGAR score, NICU admission, mechanical ventilation, RT-PCR test outcomes, feeding	Yes: 9/10
Hantoushzadeh et al.^43^ Iran	February –March 2020	Case series	n=9 25–49	Symptoms, comorbidities, diagnostic test, treatment protocol	Gestational period (weeks), maternal death, mode of delivery	Birth weight, low birth weight, 1 and 5 min APGAR score, RT-PCR test outcomes, neonatal pneumonia, intrauterine and postpartum infant death	Yes: 10/10
Hirshberg et al.^21^ United States	-	Case series	n=5 27, 29, 33, 35, 39	Symptoms, comorbidities, diagnostic test, treatment protocol	Gestational period (weeks), preterm delivery, mode of delivery, Acute Respiratory Distress Syndrome, ICU admission, mechanical ventilation	Birth weight, low birth weight, 1 and 5 min APGAR score, RT-PCR test outcomes	Yes: 9/10
Iqbal et al.^22^ United States	-	Case report	n:1 34	Symptoms, diagnostic test	Gestational period (weeks), mode of delivery, explanations of the care process	1 and 5 min APGAR score, feeding	Yes: 7/8
Juusela et al.^44^ United States	March 2020	Case series	n=2 26, 45	Symptoms, comorbidities, diagnostic test, treatment protocol	Gestational period (weeks), pregnancy-related disorders, mode of delivery explanations of the care process, postpartum health status	Fetal tachycardia	Yes: 9/10
Kalafat et al.^60^ United States	19–29 March 2020	Case report	n=1 32	Symptoms, comorbidities, diagnostic test, treatment protocol	Gestational period (weeks), preterm delivery, mode of delivery, explanations of the care process, postpartum health status	Birth weight, 1 and 5 min APGAR score, RT-PCR test outcomes	Yes: 7/8
Karami et al.^23^ Iran	-	Case report	n=1 27	Symptoms, diagnostic test, treatment protocol	Gestational period (weeks), mode of delivery, ICU admission, explanations of the care process, maternal death	1 and 5 min APGAR score, intrauterine death	Yes: 7/8
Khan et al.^45^ China	28 January – 1 March 2020	Case series	n=3 27, 28, 33	Symptoms, diagnostic test, treatment protocol	Gestational period (weeks), preterm delivery, mode of delivery, explanations of the care process	Birth weight, 1 and 5 min APGAR score, NICU admission, RT-PCR test outcomes, neonatal death	Yes: 9/10
Khan et al.^46^ China	25 January – 15 February 2020	Case series	n=17 Mean: 29.29 (range: 24–34)	Symptoms, diagnostic test, treatment protocol	Gestational period (weeks), preterm delivery, mode of delivery	Birth weight, low birth weight, 1 and 5 min APGAR score, RT-PCR test outcomes, neonatal pneumonia, neonatal death	Yes: 10/10
Koumoutsea et al.^24^ Canada	-	Case series	n=2 23, 40	Symptoms, diagnostic test, treatment protocol	Gestational period (weeks), mode of delivery, postpartum bleeding, explanations of the care process	Birth weight, low birth weight, 1 and 5 min APGAR score, fetal bradycardia, feeding	Yes: 9/10
Lee et al.^61^ Korea	14 February 2020	Case report	n=1 28	Symptoms, diagnostic test	Gestational period (weeks), preterm delivery, mode of delivery, cephalopelvic disproportion, explanations of the care process	Birth weight, 1 and 5 min APGAR score, NICU admission, RT-PCR test outcomes, feeding	Yes: 6/8
Li et al.^30^ China	24 January – 29 February 2020	Case-control	n=16 Mean: 30.9±3.2	Symptoms, comorbidities, diagnostic test, treatment protocol	Gestational period (weeks), preterm delivery, mode of delivery, pregnancy-related disorders, ICU admission, explanations of the care process, postpartum health status	Birth weight, low birth weight, 1 and 5 min APGAR score, fetal distress, RT-PCR test outcomes	Yes: 7/10
Li et al.^62^ China	6 February 2020	Case report	n=1 30	Symptoms, diagnostic test, treatment protocol	Gestational period (weeks), mode of delivery, explanations of the care process	RT-PCR test outcomes	Yes: 7/8
Li et al.^63^ China	28 January 2020	Case report	n=1 31	Symptoms, comorbidities, diagnostic test, treatment protocol	Gestational period (weeks), mode of delivery	Premature, neonatal death	Yes: 7/8
Liao et al.^47^ China	20 January – 2 March 2020	Case-control	n=10 27, 29 (n=2), 30 (n=2), 33 (n=2), 36 (n=3)	Symptoms, diagnostic test	Gestational period (weeks), preterm delivery, mode of delivery, pregnancy-related disorders, data on birth, explanations of the care process	Birth weight, premature, 1 and 5 min APGAR score, fetal distress, RT-PCR test and chest radiograph outcomes, hyaline membrane disease, neonatal asphyxia	Yes: 7/10
Liu et al.^48^ China	20 January –10 February 2020	Prospective/cross-sectional	n=15 Mean: 32±5 (range: 23–40)	Symptoms, comorbidities, diagnostic test, treatment protocol	Gestational period (weeks), mode of delivery, pregnancy-related disorders, explanations of the care process, postpartum health status	1 and 5 min APGAR score, fetal distress, neonatal asphyxia, neonatal death, stillbirth	Yes: 6/8
Liu et al.^49^ China	31 January – 29 February 2020	Prospective/cross-sectional	n=19 Median: 31 (range: 27–34)	Symptoms, diagnostic test, treatment protocol	Gestational period (weeks), mode of delivery, pregnancy-related disorders, explanations of the care process	Birth weight, 1 and 5 min APGAR score, fetal distress, NICU admission, RT-PCR test and chest radiograph outcomes, feeding	Yes: 6/8
Liu et al.^50^ China	2–5 February 2020	Case series	n=3 30 (n=2), 34	Symptoms, comorbidities, diagnostic test, treatment protocol	Gestational period (weeks), mode of delivery, explanations of the care process	Birth weight, 1 and 5 min APGAR score, fetal distress, meconium aspiration NICU admission, RT-PCR test outcomes, feeding	Yes: 9/10
Liu et al.^51^ China	8 December 2019 – 25 February 2020	Case series	n=13 22, 24, 26, 28, 29, 30 (n=3), 31, 32, 33, 35, 36	Symptoms, diagnostic test	Gestational period (weeks), preterm labor, mode of delivery, pregnancy-related disorders, ICU admission, mechanical ventilation, postpartum health status, explanations of the care process	1 and 5 min APGAR score, fetal distress, stillbirth, RT-PCR test outcomes	Yes: 9/10
Lowe and Bopp^25^ Australia	-	Case report	n=1 31	Symptoms, diagnostic test, treatment protocol	Gestational period (weeks), mode of delivery, explanations of the care process	1 and 5 min APGAR score, RT-PCR test outcomes, feeding	Yes: 7/8
Lu et al.^64^ China	11–27 February 2020	Case report	n=1 22	Symptoms, diagnostic test, treatment protocol	Gestational period (weeks), mode of delivery, postpartum health status, explanations of the care process	Birth weight, 1 and 5 min APGAR score, fetal distress, RT-PCR and Chest CT test outcomes, feeding	Yes: 7/8
Lyra et al.^26^ Portugal	-	Case report	n=1 35	Symptoms, diagnostic test	Gestational period (weeks), mode of delivery, pregnancy-related disorders, explanations of the care process	Birth weight, 1 and 5 min APGAR score, RT-PCR test outcomes	Yes: 7/8
Martinelli et al.^28^ Italy	29 March – 14 April 2020	Case report	n=1 17	Symptoms, comorbidities, diagnostic test, treatment protocol	Gestational period (weeks), preterm labor, mode of delivery, caesarean indication	Birth weight, low birth weight, NICU admission	Yes: 7/8
Peng et al.^27^ China	-	Case report	n=1 25	Symptoms, diagnostic test, treatment protocol	Gestational period (weeks), mode of delivery	Birth weight, 1 and 5 min APGAR score, fetal distress, NICU admission, RT-PCR test results	Yes: 7/8
Qiancheng et al.^31^ China	15 January – 15 March 2020	Case-control	n=28 Median: 30 (range: 26.75–32.00)	Symptoms, comorbidities, diagnostic test, treatment protocol	Gestational period (weeks), preterm labor, mode of delivery, medical abortion, explanations of the care process	Birth weight, low birth weight, 1 and 5 min APGAR score, neonatal asphyxia, NICU admission, RT-PCR test outcomes, intrauterine death, postpartum infant death	Yes: 7/10
Song et al.^65^ China	6 February 2020	Case report	n=1 30	Symptoms, diagnostic test, treatment protocol	Gestational period (weeks), mode of delivery	Birth weight, 1 and 5 min APGAR score, RT-PCR test outcomes, feeding	Yes: 7/8
Wang et al.^66^ China	1–18 February 2020	Case report	n=1 34	Symptoms, comorbidities, diagnostic test, treatment protocol	Gestational period (weeks), preterm labor, mode of delivery, pregnancy-related disorders, amniotic fluid with meconium, postpartum health status	Birth weight, 1 and 5 min APGAR score, RT-PCR test outcomes, feeding	Yes: 7/8
Wang et al.^67^ China	2–18 February 2020	Case report	n=1 28	Symptoms, diagnostic test, treatment protocol	Gestational period (weeks), preterm labor, mode of delivery pregnancy-related disorders, ICU admission	Birth weight, 1 and 5 min APGAR score, NICU admission, RT-PCR test outcomes, feeding	Yes: 7/8
Wen et al.^68^ China	4–20 February 2020	Case report	n=1 31	Symptoms, diagnostic test, treatment protocol	Gestational period (weeks), pregnancy continues	-	Yes: 7/8
Wu et al.^52^ China	23 January – 10 February 2020	Retrospective	n=8 26, 28, 29, 30 (n=3), 31, 35	Symptoms, diagnostic test, treatment protocol	Gestational period (weeks), preterm labor, mode of delivery pregnancy-related disorders, ICU admission caesarean indications, postpartum health status	Fetal distress	Yes: 6/8
Wu et al.^53^ China	31 December 2019 – 7 March 2020	Retrospective	n=23 Median: 29 (range: 21–37)	Symptoms, comorbidities, diagnostic test	Gestational period (weeks), mode of delivery pregnancy-related disorders, explanations of the care process	Birth weight, 1 and 5 min APGAR score, fetal hypoxia, RT-PCR test outcomes, clinical diagnostic criteria	Yes: 6/8
Xiong et al.^69^ China	29 January – 10 March 2020	Case report	n=1 25	Symptoms, diagnostic test, treatment protocol	Gestational period (weeks), mode of delivery pregnancy-related disorders	Birth weight, 1 and 5 min APGAR score, fetal distress, IgG, IgM and RT-PCR test outcomes	Yes: 7/8
Xu et al.^54^ China	21 January – 9 February 2020	Retrospective	n=5 23, 25, 28, 34 (n=2)	Symptoms, diagnostic test, treatment protocol	Gestational period (weeks), preterm labor, mode of delivery, pregnancy-related disorders, caesarean indications, postpartum health status, explanations of the care process	Birth weight, 1 and 5 min APGAR score, fetal distress, NICU admission, RT-PCR test outcomes, intrauterine death, postpartum infant	Yes: 6/8
Yang et al.^32^ China	20 January – 5 March 2020	Case-control	n=13 Mean: 30.2±2.3	Symptoms	Gestational period (weeks), preterm labor, mode of delivery, pregnancy-related disorders,		Yes: 7/10
Yang et al.^33^ China	20–29 January 2020	Case series	n=7	Symptoms, diagnostic test, treatment protocol	Gestational period (weeks), preterm labor, mode of delivery, pregnancy-related disorders, caesarean indications, explanations of the care process	Birth weight, 1and 5 min APGAR score, NICU admission, RT-PCR test outcomes	Yes: 1/10
Yu et al.^55^ China	1 January – 8 February 2020	Case series	n=7 29, 30, 31, 33, 34 (n=3)	Symptoms, comorbidities, diagnostic test, treatment protocol	Gestational period (weeks), mode of delivery, ICU admission, explanations of the care process	Birth weight, low birth weight, 1 and 5 min APGAR score, RT-PCR test outcomes	Yes: 10/10
Zamaniyan et al.^70^ Iran	7–26 March 2020	Case report	n=1 22	Symptoms, comorbidities, diagnostic test, treatment protocol	Gestational period (weeks), preterm labor, mode of delivery, ICU admission, maternal death, explanations of the care process	Birth weight, low birth weight, 1 and 5 min APGAR score, postpartum fever, RT-PCR test outcomes, feeding	Yes: 7/8
Zambrano et al.^71^ Honduras	9 March 2020	Case report	n=1 41	Symptoms, comorbidities, diagnostic test	Gestational period (weeks), preterm labor, mode of delivery, explanations of the care process	Birth weight, low birth weight, fetal anomaly, RT-PCR test outcomes	Yes: 7/8
Zeng et al.^34^ China	16 February – 6 March 2020	Retrospective	n=6	Symptoms, diagnostic test	Gestational period (weeks), mode of delivery, explanations of the care process	1 and 5 min APGAR score, IgG, IgM and RT-PCR test outcomes	Yes: 6/8
Zeng et al.^35^ China	January –February 2020	Retrospective	n=33	Symptoms, diagnostic test	Gestational period (weeks), preterm labor, mode of delivery, caesarean indications, pregnancy-related disorders, amniotic fluid with meconium, ICU admission, postpartum health status	Birth weight, prematurity, 1 and 5 min APGAR score, fetal distress, fetal asphyxia, NICU admission, intrauterine death, postpartum infant death, RT-PCR test outcomes, treatment protocol, mechanical ventilation	Yes: 6/8
Zhu et al.^36^ China	20 January – February 2020	Retrospective	n=9 (Twins: 1) 25, 29, 30 (n=4), 34, 35 (n=2)	Symptoms, comorbidities, diagnostic test, treatment protocol	Gestational period (weeks), preterm labor, mode of delivery, explanations of the care process	Birth weight, low birth weight, prematurity, 1 and 5 min APGAR score, fetal distress, RT-PCR test and chest radiograph outcomes, diet, postpartum infant death	Yes: 6/8

*Two of the women were still pregnant women. **In this section, those who tested and reported their results were taken.

### Methodological quality evaluation of the studies

The checklists drawn up by the Joanna Briggs Institute for cross-sectional studies, case studies, case series and case-control studies were used in the quality assessment of the articles^17^. The first and second of these tools comprise eight items, while the third and fourth comprise 10 items. Each item is assessed as Yes, No, Uncertain and Inapplicable. The results of the assessment were presented on the basis of the total number of items considered (number of Yes) as a ‘*Quality Score*’, given in Table 2.

### Synthesis of the data

The data in this systematic review and meta-analysis were combined with a meta-analysis and a consolidated percentage calculation. The data obtained from the studies of retrospective and perspective cross-sectional, case series and case-control design were consolidated in the meta-analysis. The data from the case reports were consolidated by pooled analysis. The Comprehensive Meta-Analysis Version 3-Free Trial (https://www.meta-analysis.com/pages/demo.php) was used for the meta-analysis. The extent of heterogeneity in the studies was assessed with the Cochran Q and Higgins I² tests and it was agreed that the I² rate exceeding 50% was an important indication of heterogeneity. Accordingly, when I² was greater than 50%, the Random effect results, and if the value was less, the Fixed Effect results were considered. The study data were composed of categorical variables and combined point estimates were calculated at a confidence interval (CI) of 95% for each result variable. All of the tests were calculated on a two-tailed basis and p<0.05 was accepted as statistically significant.

## RESULTS

### Searching results

At the beginning, the results of the scan yielded 732 records from the databases and 33 from additional scans, a total of 765 records. With the exclusion of the records that were repeats, the review carried out according to headings and abstracts yielded the full texts of 60 articles. From the review of the full texts, a total of 54 articles reporting the outcomes of pregnant women with COVID-19 and their infants were selected for the analysis (Figure 1).

### Study characteristics

All of the studies (n=54) had been published in English. Twenty-two of the studies were case presentations, 13 were case series, 15 were retrospective (13) and perspective (2) cross-sectional studies, and 4 were case-control. It was noted that the study data had been collected over the period 8 December 2019 – 22 April 2020 and all were published in 2020. In 10 of the studies, no indication had been given as to the date of data collection^18–27^. The studies were from 15 different countries: China (34), United States (4), Iran (4), Italy (3), Peru (1), Columbia (1), Sweden (1), United Kingdom (1), Korea (1), Canada (1), Australia (1), Portugal (1), Turkey (1) and the Netherlands (1) (Table 2).

The studies contained data on 517 pregnant women with COVID-19 and 385 infants. The sample size of the studies varied between 1–118. It was seen that the age range of the pregnant women with COVID-19 included in the systematic review was generally between 21–45, that only one women was an adolescent (age 17 years)^28^, and that seven studies did not specify the ages of the pregnant women^29–35^.

### Quality assessment results of the studies

The quality assessment scores in the case reports were Yes: 7/8 in 21 studies, and Yes: 6/8 in one study. The quality assessment scores in the case series were Yes: 10/10 in three studies and 9/10 in 11 studies. In all of the retrospective (13) and perspective (2) cross-sectional studies, the quality scores were Yes: 6/8, and in all of the case-control studies, the quality assessment scores were Yes: 7/10 (Table 2). A large majority of the studies reviewed met the criteria for quality assessment, representing a low risk of bias.

### Results of the meta-analysis

*COVID-19 symptoms in pregnant women*

The pooled results of the studies reviewed in this systematic review and meta-analysis indicated that the symptoms of the pregnant women were observed to be high fever in 53% in 29 of the studies^19,21,24,29-33,35,36,38-55^ (Z=0.503, p=0.615) and cough in 40% in 28 studies^19,21,24,29-31,33,35,36,38-43,45-56^ (Z= -1.824, p=0.068). In fifteen studies, 21% of the pregnant women with COVID-19 had fatigue/tiredness/myalgia^21,29,31,37-43,47,48,51,54,56^ (Z= -9.034, p<0.001). Nineteen studies indicated that 21% of the pregnant women with COVID-19 suffered from dyspnea/shortness of breath^19,21,29-31,38-44,46-48,51,54-56^ (Z= -4.755, p<0.001) and 16% showed the symptom of sore throat in eight studies^30,36-38,42,47,48,54^ (Z= -4.613, p<0.001). In four studies, 16% of the pregnant women with COVID-19 had headache^21,29,39,40^ (Z= -2.954, p=0.003), 15% had chest pain/tightness in seven studies^29,37,38,40,45,47,54^ (Z= -8.496, p<0.001), and 13% had phlegm in three studies^46,54,56^ (Z=-3.028, p=0.002). In twelve studies, 8% of the pregnant women with COVID-19 complained of diarrhea^29,33,36-39,41,46-49,55^ (Z= -10.701, p<0.001), while 15% suffered from runny/congested nose in six studies^21,42,46,52,56,57^ (Z= -4.177, p<0.001). Three studies indicated that 12% of the pregnant women had chills/shivers^37,38,57^ (Z= -2.366, p=0.018), one study reported that 7 of 9 pregnant women had anosmia^42^ (Z=1.562, p=0.118), and another reported that 6 of 9 pregnant women complained of lethargy^42^ (Z=0.980, p=0.327). Furthermore, according to the pooled results of four studies, about 35% of the cases had been observed to be asymptomatic, but it was seen that this result was not statistically significant^39,48,52,53^ (Z= -0.568, p=0.570) (Table 3 and Supplementary file Figure 1).

**Table 3:** Meta-analysis results of maternal health

*Outcome variables*	*Number of studies*	*Cases/total sample size*	*Point estimate (95% CI)*	*I^2^ (%)*	*Z / p*
**Symptom**					
Fever	29	252/486	53 (0.42–0.64)	72	0.503 / 0.615
Cough	28	213/474	40 (0.31–0.51)	66	-1.824 / 0.068
Dyspnea/shortness of breath	19	53/359	21 (0.13–0.31)	58	-4.755 / <0.001
Tiredness/fatigue/myalgia	15	62/325	21 (0.17–0.26)	29	-9.034 / <0.001
Diarrhea	12	21/306	8 (0.05–0.12)	0	-10.701 / <0.001
Sore throat	8	6/61	16 (0.09–0.28)	22	-4.613 / <0.001
Chest tightness/pain	7	29/198	15 (0.11–0.21)	0	-8.496 / <0.001
Runny/congestion nose	6	6/61	15 (0.03–0.42)	27	-4.177 / <0.001
Headache	4	18/170	16 (0.06–0.37)	70	-2.954 / 0.003
Phlegm	3	3/27	13 (0.04–0.34)	0	-3.028 / 0.002
Chills/shivering	3	1/21	12 (0.03–0.42)	32	-2.366 / 0.018
Anosmia	1	7/9	78 (0.42–0.94)	NA	1.562 / 0.118
Lethargy	1	6/9	67 (0.33–0.89)	NA	0.980 / 0.327
Asymptomatic	4	30/164	35 (0.05–0.83)	93	-0.568 / 0.570
**Diagnostic tests**					
PCR (+)	31	419/469	80 (0.75–0.84)	39	9.424 / <0.001
Abnormal chest CT (+)	21	285/341	80 (0.77–0.94)	56	8.097 / <0.001
Abnormal chest X-ray (+)	3	5/13	37 (0.15–0.67)	30	-0.813 / 0.416
Clinical findings	4	33/91	38 (0.19–0.61)	69	-1.029 / 0.303
**Treatments**					
Antibiotics	15	125/171	87 (0.68–0.96)	72	3.236 / 0.001
Antivirals	14	106/150	75 (0.59–0.87)	60	2.866 / 0.004
Hydroxychloroquine	5	16/61	54 (0.12–0.91)	81	0.141 / 0.888
Corticosteroids	6	12/56	31 (0.11–0.62)	58	-1.227 / 0.220
Chinese medicine (lianhua-qingwen)	5	40/44	88 (0.74–0.95)	0	4.085 / <0.001
Intravenous immunoglobulin	2	4/37	11 (0.04–0.26)	0	-3.985 / <0.001
Anticoagulant	1	8/9	89 (0.50–0.99)	NA	1.961 / 0.050
Outpatient treatment/monitoring by phone	2	32/53	77 (0.16–0.98)	76	0.829 / 0.407
Oxygen support	9	38/118	63 (0.27–0.89)	78	0.682 / 0.495
Intubation/mechanical ventilation	4	7/144	11 (0.01–0.67)	81	-1.450 / 0.147
**Treatments to prevent premature birth**					
Hydration	2	3/45	15 (0.01–0.76)	72	-1.177 / 0.239
Steroid	2	6/7	76 (0.30–0.96)	27	1.123 / 0.262
MgSO_4_	1	1/2	50 (0.06–0.94)	NA	0.000 / 1.000
**Comorbidities**					
Asthma	4	11/59	19 (0.11–0.31)	0	-4.261 / <0.001
Diabetes mellitus	3	5/57	9 (0.04–0.21)	0	-4.813 / <0.001
Hypothyroidism	5	6/70	10 (0.05–0.21)	0	-4.962 / <0.001
Chronic hypertension	3	8/64	18 (0.04–0.54)	74	-1.765 / 0.078
Obesity	4	6/18	34 (0.16–0.59)	0	-1.280 / 0.201
Heart disease	2	3/17	34 (0.01–0.97)	81	-0.317 / 0.751
Hepatitis B	3	6/67	9 (0.04–0.19)	0	-5.347 / <0.001
Polycystic ovary syndrome	3	4/25	17 (0.06–0.38)	0	-2.803 / 0.005
Thalassemia	1	1/15	7 (0.01–0.35)	NA	-2.550 / 0.011
Cholecystitis	1	1/4	25 (0.03–0.76)	NA	-0.951 / 0.341
Chronic kidney disease	1	1/5	20 (0.03–0.69)	NA	-1.240 / 0.215
Familial neutropenia	1	1/2	50 (0.06–0.94)	NA	0.000 / 1.000
**Diseases related to pregnancy**					
Gestational diabetes mellitus	11	21/181	15 (0.10–0.22)	16	-7.792 / <0.001
Hypertension	6	8/114	9 (0.05–0.18)	18	-5.922 / <0.001
Preeclampsia	7	10/57	20 (0.11–0.34)	0	-3.749 / <0.001
Cholestasis	1	1/43	2 (0.00–0.15)	NA	-3.694 / <0.001
Anemia	2	2/14	16 (0.04–0.46)	0	-2.181 / 0.029
Hypothyroidism	1	1/10	10 (0.01–0.47)	NA	-2.084 / 0.037
Decrease in fetal movement	2	2/47	8 (0.01–0.53)	66	-1.879 / 0.060
Multiple pregnancy	1	2/118	2 (0.00–0.07)	NA	-5.693 / <0.001
Early membrane rupture	8	20/132	17 (0.11–0.25)	18	-6.260 / <0.001
Placental abruption	4	4/40	11 (0.04–0.25)	0	-3.980 / <0.001
Placenta previa	4	4/33	14 (0.05–0.31)	0	-3.400 / 0.001
Polyhydramnios/oligohydramnios	2	3/14	21 (0.07–0.50)	0	-1.992 / 0.046
Postpartum bleeding	1	1/2	50 (0.06–0.94)	NA	0.000 / 1.000
**Admission to ICU**	8	14/162	12 (0.04–0.33)	65	-3.039 / 0.002
**Maternal death**	2	7/127	12 (0.00–0.99)	94	-0.597 / 0.550
**Duration of pregnancy and mode of delivery**					
Caesarean delivery	30	314/405	73 (0.68–0.78)	44	7.979 / <0.001
Indication of COVID-19 for caesarean section	5	56/166	29 (0.14–0.51)	78	-1.872 / 0.061
Indication of obstetric for caesarean section	8	63/176	43 (0.28–0.60)	66	-0.837 / 0.403
Preterm labor (<37 weeks)	17	60/348	25 (0.17–0.35)	55	-4.304 / <0.001
**COVID-19 test results in postpartum period**					
**The first 24 hours**					
Placenta and umbilical cord blood samples (+)	8	1/43	11 (0.04–0.27)	0	-3.916 / <0.001
Breast milk (+)	6	0/35	9 (0.03–0.26)	0	-3.691 / <0.001
Vaginal swab (+)	2	0/5	14 (0.02–0.58)	0	-1.647 / 0.100
Amniotic fluid (+)	7	0/42	9 (0.03–0.22)	0	-4.158 / <0.001
2nd–14th day					
Breast milk (+)	3	2/7	33 (0.09–0.70)	0	-0.900 / 0.368
Vaginal swab (+)	1	0/2	17 (0.01–0.81)	NA	-1.039 / 0.299

*Diagnostic test results for COVID-19 in pregnant women*

According to the pooled results of 31 studies, 80% of the pregnant women tested positive on the RT-PCR test^19,21,24,29,30,32-57^ (Z=9.424, p<0.001), and 88% had abnormal chest CTs in 21 studies^30,32-40,43,46-50,52-54,56,57^ (Z=8.097, p<0.001). In three studies, 37% of the women had abnormal chest X-rays^24,42,44^ (Z= -0.813, p=0.416). Moreover, in the pooled results of four studies^29,34,49,52^, 38% of the pregnant women had been diagnosed on the basis of clinical findings (Z= -1.029, p=0.303) (Table 3 and Supplementary file Figure 2).

*Treatment methods using for COVID-19 in pregnant women*

Fifteen studies taken into the meta-analysis showed that 87% of the pregnant women were treated with antibiotics^29-31,33,38,43-46,48,50,52,54,55^ (Z=3.236, p=0.001), and 75% were given antiviral treatment in 14 studies^19,21,30,31,38,43,45,46,48-50,54,55,57^ (Z=2.866, p=0.004, I^2^=60%). In five studies, 54% of the pregnant women were treated with hydroxychloroquine^19,21,29,43,44^ (Z=0.141, p=0.888), while 31% were treated with corticosteroids in six studies^31,36,38,50,54,57^ (Z= -1.227, p=0.220). In five studies, 88% of the pregnant women were treated with a Chinese medication (lianhua-gingwen)^45,46,48,55,57^ (Z=4.085, p<0.001). In two studies, 11% of the pregnant women were given intravenous immunoglobulin^31,43^ (Z= -3.985, p<0.001) and 8 of 9 pregnant women were given anticoagulant treatment in one study^43^ (Z=1.961, p=0.050). Again, 63% of the pregnant women needed oxygen support in 9 studies^29,33,38,41,44,45,50,54,55^ (Z=0.682, p=0.495), and 11% were intubated/placed on mechanical ventilation in 4 studies^21,39,51,52^ (Z= -1.450, p=0.147). According to the results of two studies, 77% of the pregnant women with COVID-19 had been treated and followed up as outpatients/by telephone^29,37^ (Z=0.829, p=0.407) (Table 3 and Supplementary file Figure 3).

This meta-analysis showed that in 2 studies it was reported that in order to prevent preterm labor, 15% were treated with hydration^29,44^ (Z= -1.177, p=0.239), and 6 of 7 pregnant women were given steroids in another two studies^21,44^ (Z=1.123, p=0.262), and 1 of 2 pregnant women were treated with magnesium sulphate (MgSO_4_) in one other study^44^ (Table 3 and Supplementary file Figure 4).

*Comorbidities of pregnant women with COVID-19*

The results of the meta-analysis indicated that 19% of the pregnant women in 4 studies had asthma^21,24,29,42^ (Z= -4.261, p<0.001), and it was reported in 3 studies that 18% of the women suffered from chronic hypertension^21,29,30^ (Z= -1.765, p=0.078). It was reported in 5 studies that 10% of the pregnant women had hypothyroidism^31,43,50,53,55^ (Z= -4.962, p<0.001), and 9% of the pregnant women had diabetes mellitus in three studies^21,29,43^ (Z= -4.813, p<0.001). It was found in 4 studies that 34% were obese^21,24,43,44^ (Z= -1.280, p=0.201), 34% had heart disease in two studies^44,48^ (Z= -0.317, p=0.751). It was reported in three studies that 9% of the pregnant women had Hepatitis B^30,31,53^ (Z= -5.347, p<0.001) and 17% of the women had polycystic ovary syndrome in three studies^30,44,55^ (Z= -2.803, p=0.005). In one study, 7% of the pregnant women were found to have thalassemia^48^ (Z= -2.550, p=0.011), and 1 of 4 of the pregnant women had cholecystitis in another study^40^ (Z= -0.951, p=0.341). It was noted in one study^21^ that 1 of 5 pregnant women had chronic kidney disease (Z= -1.240, p=0.2015), and 1 of 2 pregnant women had familial neutropenia in another study^24^ (Table 3 and Supplementary file Figure 5).

*Additional conditions related to pregnancy*

The pooled results of 11 studies in the meta-analysis showed that about 15% of the pregnant women had gestational diabetes mellitus^24,29-31,37,41-44,48,56^ (Z= -7.792, p<0.001), 6 studies indicated that 9% had hypertension^29,31,38,42,44,53^ (Z= -5.922, p<0.001), and 7 studies reported that 20% had preeclampsia^30,33,37,38,44,52,56^ (Z= -3.749, p<0.001). It was reported in one study that 2% of the pregnant women had cholestasis^29^ (Z= -3.694, p<0.001) and 16% of the pregnant women had anemia in another 2 studies^37,40^ (Z= -2.181, p=0.029). Another study revealed that 10% of the pregnant women had hypothyroidism^37^ (Z= -2.084, p=0.037) (Table 3 and Supplementary file Figure 6).

In another 2 studies, 8% of the pregnant women with COVID-19 exhibited reduced fetal movement^29,40^ (Z= -1.879, p=0.060), 2% of the pregnant women were in multiple pregnancy in another study^39^ (Z= -5.693, p<0.001), and 17% had early membrane rupture in 8 studies^30,35-38,49,51,53^ (Z= -6.260, p<0.001). It was reported that 11% of the pregnant women in 4 studies had placental abruption^30,36,37,54^ (Z= -3.980, p<0.001), 14% of the pregnant women in four studies had placenta previa^36,40,48,54^ (Z= -3.400, p=0.01), and 21% of the pregnant women in 2 studies had polyhydramnios/oligohydramnios^36,54^ (Z= -1.992, p=0.046) (Table 3 and Supplementary file Figure 6).

The pooled results in this meta-analysis showed that 8 studies indicated that 12% of the pregnant women diagnosed with COVID-19 were admitted into the intensive care unit^21,29,30,35,41,44,51,52^ (Z= -3.039, p=0.002), and 12% of the pregnant women in 2 studies were recorded as maternal mortalities^39,43^ (Z= -0.597, p=0.550). In one other study, it was noted that one of the two pregnant women had postpartum hemorrhage^24^. While the postpartum hemorrhage and maternal death results pertaining to the pregnant women with COVID-19 were not statistically significant, data related to admittance into the intensive care unit was statistically significant (Table 3 and Supplementary file Figures 7 and 8).

*Results on pregnancy term and mode of delivery in pregnant women*

Thirty studies examined in this systematic review and meta-analysis provided results on the delivery mode of pregnant women with COVID-19^19,21,24,29-44,46,48-57^. The pooled results of these studies were statistically significant in that 73% of the pregnant women with COVID-19 had delivered by caesarean section (Z=7.979, p<0.001). According to the results of 5 studies, 29% of the women undergoing caesarean section had a COVID-19 indication^35,39,41,42,54^ (Z= -1.872, p=0.061), and 43% of the pregnant women in 8 studies had undergone caesarean section due to obstetric indications^29,33,37,39,41,42,52,54^ (Z= -0.837, p=0.403). These results, however, were not statistically significant. It was found that 25% of the pregnant women in 17 studies went into preterm labor and this result was statistical significance^21,29-33,35-39,42,45-47,51,54^ (Z= -4.304, p<0.001) (Table 3 and Supplementary file Figure 9).

*Perinatal results of newborns*

In 15 studies examined in this systematic review and meta-analysis, 25% of the newborns of women with COVID-19 were born with low birth weight (<2500 g)^19-21,24,30,31,35-38,42,43,46,54,55^ (Z= -4.772, p=0.001). It was found that about 11% of the newborns in 26 studies had an APGAR score of <7 in the first minute^19-21,24,29-31,33-40,42,43,45,46,48,50,51,53-55,57^ (Z= -8.527, p<0.001). It was determined that 9% of the newborns had an APGAR score of <7 in the fifth minute in 27 studies^19-21,24,29-31,33-38,40-43,45,46,48,50,51,53,54,55-57^ (Z= -9.490, p<0.001). It was revealed that 23% of the newborns suffered fetal distress in 13 studies^19,24,30,35-38,44,48-52^ (Z= -4.591, p<0.001), and 4% of the newborns had fetal asphyxia in 10 studies^31,35-39,47,48,53,55^ (Z= -8.078, p<0.001) (Table 4 and Supplementary file Figure 10).

**Table 4:** Meta-analysis results on newborn health

*Outcome variables*	*Number of studies*	*Cases/total sample size*	*Point estimate (95% CI)*	*I^2^ (%)*	*Z / p*
**Perinatal outcomes**					
Low birth weight (<2500 g)	15	31/162	24 (0.140–0.386)	51	-4.772 / 0.001
Apgar Score in the first minute <7	26	12/317	11 (0.072–0.167)	18	-8.527< / <0.001
Apgar Score in the fifth minute <7	27	12/594	9 (0.061–0.142)	0	-9.490 / <0.001
Fetal distress	13	21/136	23 (0.230–0.333)	40	-4.591 / <0.001
Fetal asphyxia	10	3/198	4 (0.020–0.086)	0	-8.078 / <0.001
Admission to ICU of newborn	11	40/168	28 (0.128–0.515)	70	-4.993 / 0.066
**Neonatal death**	12	4/216	6 (0.033–0.122)	0	-7.411 / <0.001
**Intrauterine death**	5	2/84	8 (0.029–0.198)	22	-4.564 / <0.001
**Feeding and care**					
Breastfeeding	9	33/85	36 (0.148–0.646)	53	-2.911 / 0.338
Formula	3	8/8	86 (0.521–0.972)	0	2.057 / 0.040
Isolated separate from mother	14	87/90	88 (0.78–0.94)	0	5.191 / 0.000
Isolated with mother	3	32/62	77 (0.14–0.99)	83	0.779 / 0.436
**COVID-19 test outcomes**					
**The first 24 hours**					
RT-PCR (+)	26	9/278	8 (0.05–0.12)	0	-10.581 / <0.001
IgG (+)	2	2/8	29 (0.08–0.64)	0	-1.206 / 0.228
IgM (+)	2	3/8	38 (0.12–0.72)	0	-0.683 / 0.495
**2nd–14th day**					
RT-PCR (+)	8	1/61	10 (0.04–0.23)	0	-4.285 / <0.001
Abnormal chest X-ray / CT	5	12/39	31 (0.11–0.62)	58	-1.199 / 0.231

In 11 of the studies in the meta-analysis, 28% of the newborns had been admitted into the neonatal intensive care unit (NICU) and this result was statistically insignificant^20,29,31,33-35,40-42,45,49,50^ (Z= -4.993, p=0.066). According to the pooled results of 12 of the studies examined, the neonatal death was observed in 6% of newborns and this result was statistical significance^31,35-39,43,45,46,48,51,54^ (Z= -7.411, p<0.001). It was found that the intrauterine death rate was 8% in a meta-analysis based on the results of 5 studies^31,35,38,43,54^ (Z= -4.564, p<0.001) (Table 4 and Supplementary file Figures 10–12).

*Data on neonatal nutrition and care*

In 9 studies examined in this meta-analysis, 36% of the newborns were breastfed by their mothers, who wore masks and strictly complied with hand-washing rules^19,20,24,29,41,42,50,56,57^ (Z= -2.911, p=0.338), and all of the eight newborns were fed formula in three studies^19,20,40^ (Z= 2.057, p=0.040). These results were statistically significant. Again, statistical significance was noted in 14 studies regarding the data that 88% of the newborns were isolated from their mothers^19,30,32-34,40,42,44,45,47,49,50,54,57^ (Z= 5.191, p<0.001). In 3 studies, 77% of the newborns were isolated together with their mothers, but this finding was not found to be statistically significant^20,29,41^ (Z=0.779, p=0.436) (Table 4 and Supplementary file Figure 13).

*COVID-19 test outcomes of newborns*

The consolidated results of 26 studies in this meta-analysis showed that 8% of newborns of women with COVID-19 the newborns tested positive in the RT-PCT test in the first 24 hours after birth^19-21,29,31-39,41-43,45-47,49-51,53,54,56,57^ (Z= -10.581, p<0.001), in 2 studies, 2 of 8 newborns tested positive for IgG^19,34^ (Z= -1.206, p=0.228), and 3 of 8 newborns tested positive for IgM in two studies^19,34^ (Z= -0.683, p=0.495) (Table 4 and Supplementary file Figure 14).

The consolidated results of eight studies in this meta-analysis revealed that 10% of the newborns tested positive on the RT-PCR test in the 48th hour and on day 14 postpartum^19,20,30,31,33,37,40,47^ (Z= -4.285, p<0.001). It was also seen in another 5 studies^36,40,47,49,57^ that 31% recorded abnormal results on the chest X-ray/CT (Z= -1.199, p=0.231) (Table 4 and Supplementary file Figure 15).

*COVID-19 test outcomes in placenta, umbilical blood, breast milk, vaginal swab and amniotic fluid samples*

In 8 studies examined, one of the placenta and the umbilical blood samples taken from pregnant women with COVID-19 in the first 24 hours postpartum was statistically significantly positive^19,33,38,45,46,50,56,57^ (Z= -3.916, p<0.001). On the other hand, samples of breast milk in 6 studies^19,38,39,49,50,57^ (Z= -3.691, p<0.001), of vaginal swab in 2 studies^50,57^ (Z= -1.647, p=0.100), and of amniotic fluid in 7 studies^19,33,38,49,50,56,57^ (Z= -4.158, p<0.001) did not test positive. In days 2–14 postpartum, 2 of 7 samples of breast milk were positive in 3 studies^19,20,55^ (Z= -0.900, p=0.368), and both of the two vaginal swabs were negative in one study^17^ (Z= -1.039, p=0.299) (Table 3 and Supplementary file Figures 16 and 17).

*Pooled analysis results from case reports*

According to the pooled results of the 22 case reports^18,20,22,23,25-28,58-71^, the most common symptoms among pregnant women recorded in case reports were fever (81.8%), cough (54.5%), fatigue/tiredness/ myalgia (40.9%) and dyspnea/shortness of breath (40.9%). It was seen that all of these pregnant women tested negative in the RT-PCR test and 72.2% had normal chest-CT scans; the method of treatment was mostly with antibiotics (68.2%) and antiviral drugs (59.1%). Of the pregnant women, 36.4% had underlying conditions and 36.4% had a history of preterm labor; 75% had delivered by caesarean. Eight mothers were admitted into intensive care and two mothers lost their lives. Six out of 20 newborns were admitted into the NICU, and only 2 were being breastfeed while 11 were separated and isolated from their mothers. It was seen that in 1 of the 13 infants tested in the first 24 hours after birth tested positive on the RT-PCR test and 1 of 3 babies tested positive for IgG and IgM. In the later period (48 hours –14 days), 2 of the 8 infants tested had positive RT-PCR results (Table 5).

**Table 5:** Pooled results of cases

*Outcome variables*	*Pooled n (%)*	*Outcome variables*	*Pooled n (%)*
**Data on maternal health**			
**Symptoms (n=22)**		**Treatments (n=22)**	
Fever	18 (81.8)	Antivirals	13 (59.1)
Chills/shivering	3 (13.6)	Antibiotics	15 (68.2)
Cough	12 (54.5)	Hydroxychloroquine	3 (13.6)
Phlegm	1 (4.5)	Corticosteroids	8 (36.5)
Sore throat	2 (9.1)	Anticoagulant	1 (4.5)
Hoarseness	1 (4.5)	Hydration	1 (4.5)
Runny/congestion nose	3 (13.6)	Tocolytic therapy	3 (13.6)
Tiredness/fatigue/myalgia	9 (40.9)	Antenatal steroid	2 (9.1)
Dyspnea/shortness of breath	9 (40.9)	Oxygen support	6 (27.3)
Headache	2 (9.1)	Intubation/mechanical ventilation	6 (27.3)
Diarrhea	1 (4.5)	Blood transfusion/iron supplement	4 (24.1)
Asymptomatic	1 (4.5)	Traditional Chinese medicine, supportive therapy	1 (4.5)
**Diagnostic tests (n=22)**		**Diseases related to pregnancy (n=22)**	
RT-PCR (+)	22 (100)	Gestational diabetes mellitus	1 (4.5)
Abnormal Chest CT (+)	16 (72.2)	Gestational hypertension	1 (4.5)
Abnormal Chest X-ray (+)	6 (27.3)	Hyperemesis gravidarum	1 (4.5)
Abnormal Chest USG	1 (4.5)	Reflux	1 (4.5)
Elevated IgG and IgM	1 (4.5)	Preeclampsia	1 (4.5)
With comorbidities (n=22)	8 (36.4)	Preterm labor (<37 weeks)	8 (36.4)
Comorbidities (n=8)		Multiple pregnancy	2 (9.1)
Diabetes Mellitus	1	Early membrane rupture	2 (9.1)
Asthma	1	Admission to ICU (n=22)	8 (36.4)
Hypothyroidism	3	Maternal death (n=22)	2 (9.1)
Migraine	1		
Obesity	1		
Thalassemia	1		
Duration of pregnancy and mode of delivery (n=20)*			
Vaginal delivery	5 (25.0)		
Caesarean delivery	15 (75.0)		
Indication of COVID-19 for caesarean section	2		
Indication of obstetric for caesarean section	1		
Data on new born health (n=20)*			
Low birth weight (<2500 g)	4	**COVID-19 test results in postpartum period**	
Apgar Score in the first minute <7	1	The first 24 hours**	
Apgar Score in the fifth minute <7	1	IgG and IgM (+)	1/3
Fetal distress	1	RT-PCR (+)	1/13
Fetal anomaly	1	2nd–14th day**	
Admission to ICU of new born	6	RT-PCR (+)	2/8
Intrauterine death	1	Abnormal chest X-ray / CT	0/2
Neonatal death	1	Placenta and umbilical cord blood samples (+)	0/6
Breastfeeding	2	Breast milk (+)	0/5
Formula	6	Vaginal swab (+)	
Isolated separate from mother	11	Amniotic fluid (+)	0/4
Isolated with mother	1		

* Two of the women were still pregnant women.

** In this section, those who tested and reported their results were taken.

## DISCUSSION

This systematic review and meta-analysis present the consolidated results of 54 observational studies, case reports and case series studies reporting the maternal and infant health outcomes during the pregnancy, birth and postpartum periods of pregnant women with COVID-19. The results of these studies are significant since they can contribute to improving the processes of treatment, follow-up and care services offered to pregnant women with COVID-19 and their newborns.

### Outcomes of pregnant women with COVID-19

This systematic review and meta-analysis revealed that pregnant women with COVID-19 most commonly complained of high fever, chills/shivers, fatigue/tiredness/myalgia, cough, dyspnea/shortness of breath, chest pain/tightness, phlegm, runny nose/congestion, diarrhea, headache and sore throat. Similar results have been reported in previous systematic reviews concerning pregnant women^5,6,72-75^. In this study, it was found that COVID-19 was asymptomatic in approximately one-third of the pregnant women (35%). Lower percentages were reported in studies by Elshafeey et al.^6^ and Mullins et al.^76^ (7.5% and 32%, respectively). The results are similar to those observed in non-pregnant women^77^ and are valuable in terms of offering data that will be useful in the early diagnosis, follow-up and care of pregnant women.

It was found in this meta-analysis that a large percentage of the pregnant women with COVID-19 tested positive on the RT-PCR (80%), revealed an abnormal chest CT (88%) and that some women displayed abnormal chest X-rays (37%). The results of some studies show that a significant percentage of pregnant women with COVID-19 (38%) were diagnosed with clinical findings. Similarly, Smith et al.^74^ also report that approximately 90% of the women in their study were administered the RT-PCR test to confirm the diagnosis, that in 79% of the cases, tests were positive and RT-PCR negative in 23%. The authors reported that 99% of the pneumonia cases were diagnosed with CT findings. De Rose et al.^5^ state that the respiratory system samples taken from the women tested positive on the RT-PCR test in almost all of the women (99%). Elshafeey et al.^6^ report, however, that most of the women (90%) were tested with RT-PCR for a diagnosis and the infection was confirmed with radiological and clinical findings in a smaller percentage of women (10%). Della Gatta et al.^72^ report similar results. These data were similar with the diagnostic tests used with non-pregnant individuals^77,78^, indicating that more information is needed regarding the effects on pregnant women and infant health of diagnostic tests carried out with radiological methods.

It was noted in this systematic review and meta-analysis that pregnant women with COVID-19 were treated with antiviral drugs, antibiotics, intravenous immunoglobulin, a Chinese drug, hydroxychloroquine, corticosteroids, anticoagulants and oxygen support. Another study similarly indicated that pregnant women with COVID-19 were commonly treated with antibiotics, oxygen, antiviral drugs and corticosteroids^73^. It was reported in another study that a significant percentage of hospitalized pregnant women (28%) were put on oxygen support^74^. These reports show that pregnant women with COVID-19 were treated similarly to non-pregnant individuals^78^. There is a need for more data on the outcomes of such treatments on pregnancy and the infant.

In 2 studies examined in this systematic review and meta-analysis, a large majority of the pregnant women with COVID-19 were treated as outpatients or followed up by telephone. Again, it was reported in some studies with respect to preterm labor that hydration, steroid and MgSO_4_ therapy was applied to some of the pregnant women. These reported results are valuable in that they indicate that pregnant women with COVID-19 can be followed up with routine care similar to what would be implemented under normal circumstances.

This meta-analysis showed that some pregnant women with COVID-19 had underlying conditions such as diabetes mellitus, asthma, chronic hypertension, hypothyroidism, Hepatitis B, obesity, heart disease and polycystic ovary syndrome. On the other hand, Della Gatta et al.^72^ report no pre-existing comorbidities before the pregnancy. It is known that COVID-19 presents a life-threatening risk when there are comorbidities^77^ and that high-risk pregnant women with a history of chronic disease must particularly be carefully protected, making follow-up a matter of vital importance.

The study revealed that some pregnant women had diabetes mellitus, hypertension, preeclampsia and anemia. In a similar study, Della Gatta et al.^72^ reported that one of the 35 pregnant women had gestational hypertension while another suffered from preeclampsia. These conditions can be life-threatening for pregnant women with COVID-19 and necessitate cautious care and follow-up.

Furthermore, the study demonstrated that some pregnant women have additional conditions during their illness such as reduced fetal movement, multiple pregnancy, early membrane rupture, placental abruption, placenta previa and polyhydramnios/oligohydramnios. A previous study^5^ revealed similar results. These conditions may mean added difficulties in treatment, follow-up and care.

### Outcomes related to the gestation period in pregnant women with COVID-19 and their mode of delivery

Our study indicates that preterm labor was observed in about 18% of the pregnant women with COVID-19. While this percentage is lower in the studies of Della Gatta et al.^72^ and Elshafeey et al.^6^ (12% and 15%, respectively), and De Rose et al.^5^, Smith et al.^74^ and Yang et al.^75^ report higher rates (34%, 64% and 21%, respectively). These rates are also higher than what is reported for the general population (11%)^79,80^. Preterm birth is closely associated with infant fatality and therefore this information must be carefully taken into consideration in the care and follow-up of infants with COVID-19.

This systematic review and meta-analysis reports that most of the pregnant women with COVID-19 (77%) were delivered by caesarean section. In some of the studies examined, caesarean childbirth was implemented mostly for obstetric indications (43%) and for some women for COVID-19 indications (29%). Elshafeey et al.^6^, De Rose et al.^5^ and Smith et al.^74^ reported similar caesarean rates in their studies (69%, 77% and 80%, respectively). It can be seen in other studies that the caesarean birth rate in women with COVID-19 is much higher^72,73,75^. Although these outcomes have not been reported as indications, it is apparent that the caesarean birth rate among pregnant women with COVID-19 is markedly high. This outcome should be assessed in terms of maternal and infant health.

### Perinatal outcomes of the newborns of pregnant women with COVID-19

It was noted in this systematic review and meta-analysis that 19% of the newborns of women with COVID-19 was of low birth weight. A previous study revealed a similar percentage^5^. Contrary to this data, however, Elshafeey et al.^6^ and Yang et al.^75^ report a lower frequency of low birth weight (8% and 5.3%, respectively) while Smith et al.^74^ point to a higher rate (43%). These results can be associated with the incidence of preterm labor among pregnant women with COVID-19.

It was found in this systematic review and meta-analysis that some infants born of pregnant women with COVID-19 had low APGAR scores (<7) in the first (4.3%) and fifth minute (4.6%), suffered from fetal asphyxia and fetal distress (14%). The incidence of fetal distress was reported as lower in studies by Elshafeey et al.^6^ and Yang et al.^75^ (8% and 11%, respectively). De Rose et al.^5^ reported this rate to be 22%. In Smith et al.^74^, it was reported that all the newborns in their study exhibited normal APGAR scores. Again, in the study by Yang et al.^75^, a similar outcome was reported with regard to neonatal asphyxia (1.2%). These results are significant in that they indicate that infants born of women with COVID-19 must be kept under close scrutiny in the perinatal period.

In some studies that were reviewed for this systematic review and meta-analysis, there were data that indicated that some newborns were being breastfed (33%). Elshafeey et al.^6^ had presented similar results in their systematic review. These data are valuable in terms of the health of breastfed infants and indicate the need for additional research and the adoption of urgent measures to develop methods by which women with COVID-19 can safely provide their infants with the breast milk they require.

It was seen in the meta-analysis that some newborns of women with COVID-19 were administered various tests in the first 24 hours and later after birth. The analysis showed that some infants tested positive in the first 24 hours and some in the period Day 2 – Day 14 on the RT-PCR test. It was also seen that some infants displayed positive IgG and IgM results in the first 24 hours. It was found in the meta-analysis that the samples taken from pregnant women with COVID-19 (placenta, umbilical blood, breast milk, vaginal swab and amniotic fluid) tested positive on the COVID-19 test in the first 24 hours in only one woman in the placenta and umbilical blood specimens and in two women in Days 2–14 in the breast milk specimens. Similar results can be seen in other studies as well^5,6,72,74^. These results are not sufficient, however, in terms of making an assessment of the possibility of vertical transmission; more evidence is needed.

### Outcomes on admittance of pregnant women with COVID-19 and their infants into the intensive care unit and maternal–infant death

It was noted in this systematic review and meta-analysis that a significant portion of the pregnant women were admitted into the intensive care unit and were administered intubation/mechanic ventilation. Elshafeey et al.^6^ similarly reported that some women were admitted into intensive care (4.4%) and were administered mechanical ventilation (1.6%). In the study by Smith et al.^74^, it was reported that one woman (4.3%) needed mechanical ventilation and was admitted into the intensive care unit. These results indicated that COVID-19 is a serious health issue that threatens maternal/infant health during the period of pregnancy and beyond.

In all of the studies reviewed for this systematic review and meta-analysis, nine mothers were reported to have lost their lives. In their study of 108 pregnant women, Zaigham and Andersson^73^ reported no incidents of maternal mortality. Although the percentage was higher than the general maternal mortality rate, it is lower that the mortality rate reported for SARS-CoV-2 (3.8%)^81^.

It was again seen in this study that a significant portion of the newborns were admitted into the NICU. Similarly, in some other previously published systematic reviews, it was reported that some infants had been admitted to the neonatal intensive care unit due to an additional symptom or for further care and follow-up^6,74^. These results may be associated with the high level of premature birth and low birth weight rates.

In all of the studies reviewed for this systematic review and meta-analysis, it was reported that 3 out of 385 infants lost their lives in the intrauterine stage and 5 died as neonates. Smith et al.^74^ also reported in the data they presented for 37 infants that one baby had died in the intrauterine and one in the neonatal stages. Moreover, Yang et al.^75^ and Zaigham and Andersson^73^ reported one mortality in the intrauterine and neonatal stages in their studies. In the systematic review of Elshafeey et al.^6^, the authors provided findings from 256 newborns, reporting two stillbirths and one neonatal death. Both De Rose et al.^5^ and Della Gatta et al.^72^ reported one stillbirth in each of their studies. The rates are higher than in general infant rates and are important in that they indicate that COVID-19 is responsible for increasing infant fatalities.

### Strengths and limitations

The high score noted in the current quality assessment of the studies examined in this systematic review and meta-analysis and the wide range of additional resources available for scanning constituted the strengths of the study. Also, the large number of pregnant women and infants whose data were reviewed in this systematic review was another strong point that consequently strengthened the conclusions that were drawn. The study was further strengthened by the fact that the results were based on reliable methods of analysis, the subject matter was examined from different aspects, and the results attained were supported by outcomes reported in previous studies. It can be said, however, that the low extent of homogeneity in the studies reviewed may have weakened the power of the evidence. To keep this factor under control, the Random Effect model was preferred in analyses in which the extent of heterogeneity was high. Other limitations were that only studies published in English were taken into the analysis, most of the studies were based on small sample sizes and were derived from Chinese sources, China being the location where COVID-19 had originated, which also meant that studies published in Chinese and other languages could not be included.

## CONCLUSIONS

We tried in this systematic review and meta-analysis to uncover comprehensive data on the status of pregnant women with COVID-19 and their infants, based on the results of 54 studies. The evidence gathered showed that the symptoms, diagnosis, treatment and comorbidity factors associated with COVID-19 were the same in pregnant women as in non-pregnant women. It was, however, noticeable that morbidity, preterm and caesarean birth rates necessitating admission to the intensive care unit as well as maternal and perinatal death rates were higher in pregnant women with COVID-19 and their infants. It was also found that some babies tested positive in the first 24 hours and on Days 2–14 in the RT-PCR, IgG and IgM tests. An important part of these results is also supported by previous systematic review and meta-analysis studies^9,10^.

Based on existing data, health professionals play a key role in protecting and improving the health of pregnant women during a pandemic. Besides tending to the needs of the other members of the community in this time, these health professionals must take preventive and risk-reducing measures in their care of the high-risk group of pregnant and possibly pregnant women to ensure that no contact is made with the SARS-CoV-2 infection. Moreover, they need to take into consideration the individual characteristics of each pregnant woman in their effort to protect and improve maternal and infant health, basing their actions on the guidelines to care and follow-up set forth by international organizations. It is necessary also in this period to take the measures that will ensure the prevention of neonatal infection and to develop guidelines that will allow infants to benefit from breast milk. Additionally, scientific studies are needed that will explore the long- and short-term effects of the infection and the impact of care and follow-up services specifically adopted for use during the pandemic on maternal and infant morbidity and mortality.

## Data Availability

Data sharing is not applicable to this article as no new data were created.

## PROTOCOL REGISTRATION

Prospero: CRD42020191106. Date of registration: 11 June 2020. Title: Maternal and perinatal conditions of pregnancy in COVID-19: systematic review and meta-analysis
